# A deep learning-based radiomic nomogram derived from visceral fat for early prediction of gastrointestinal stromal tumor risk grade

**DOI:** 10.3389/fmed.2026.1741436

**Published:** 2026-06-19

**Authors:** Wei Chen, Long-Yu Duan, Kun-Ming Yi, Xiao-Juan Peng, Lian-Qin Kuang

**Affiliations:** 1 Department of Radiology, The Third Affiliated Hospital of Chongqing Medical University, Chongqing, China; 2 Department of Radiology, Daping Hospital, Army Medical University, Chongqing, China

**Keywords:** deep learning, gastrointestinal stromal tumor, nomogram, non-contrast CT, radiomics, risk stratification, visceral adipose tissue

## Abstract

**Background:**

Gastrointestinal stromal tumors (GISTs) show substantial heterogeneity and are classified into distinct risk categories requiring different treatment strategies. Therefore, reliable preoperative risk stratification is crucial for treatment planning. Due to its inherent contrast, visceral adipose tissue can be reliably segmented and its volume of interest (VOI) obtained even on non-contrast CT scans. Therefore, this study aimed to develop a deep-learning-based radiomics nomogram (DLRN) that integrates visceral adipose features extracted from non-contrast computed tomography (CT). The DLRN is designed to provide a convenient tool for the preoperative prediction of GIST risk grades, including for patients for whom contrast-enhanced imaging is not feasible.

**Patients and methods:**

A total of 211 patients with histologically confirmed GISTs from two institutions were included. The derivation cohort from Institution A (*n* = 158) was randomly divided into a training cohort (*n* = 110) and an internal validation cohort (*n* = 48) at a 7:3 ratio. An independent external test cohort from Institution B (*n* = 53) was used for external validation. Visceral fat features were extracted from non-contrast CT images, and the DLRN was constructed for preoperative risk grading. Model performance was compared with clinical, traditional radiomics, deep learning radiomics, and feature-fusion models.

**Results:**

Among the evaluated models, the DLRN showed favorable discrimination in the derivation cohort and exploratory performance in the external test cohort. In the derivation cohort, it achieved an area under the curve (AUC) of 0.936 [95% confidence interval (CI): 0.8907–0.9812], accuracy of 0.873, sensitivity of 0.811, and specificity of 0.904. In the external test cohort, the AUC was 0.862 (95% CI: 0.6216–1.0000), with an accuracy of 0.925, a sensitivity of 0.833, and a specificity of 0.936. Decision curve analysis demonstrated that the DLRN provided a higher net clinical benefit than all comparison models in both datasets. Calibration curves showed the agreement between predicted probabilities and observed outcomes, while DeLong tests were used for pairwise AUC comparisons between models.

**Conclusion:**

The proposed DLRN, integrating visceral fat-derived radiomics and clinical variables, may provide a non-invasive adjunct for preoperative GIST risk stratification on non-contrast CT. Further validation in larger multicentre cohorts is still needed.

## 1 Introduction

Gastrointestinal stromal tumors (GISTs) originate from the interstitial cells of Cajal. Although they constitute only a small proportion of gastrointestinal malignancies, GISTs exhibit diverse biological behaviors, with certain subtypes demonstrating significant potential for recurrence and metastasis. The National Institutes of Health (NIH) criteria and the revised classification system from the Armed Forces Institute of Pathology (AFIP) are currently the most widely used risk stratification frameworks. Based on tumor size, mitotic rate, and primary site, gastrointestinal stromal tumors (GISTs) are classified into very low-risk, low-risk, intermediate-risk, and high-risk categories. According to the “Chinese Consensus on Comprehensive Management of Gastrointestinal Stromal Tumors (2020 Edition),” high-risk patients with GIST and intermediate-risk patients with non-gastric primary tumors should receive at least 3 years of imatinib adjuvant therapy. In contrast, intermediate-risk patients with gastric primary tumors are recommended to undergo at least 1 year of imatinib adjuvant therapy. Conversely, very low-risk and low-risk patients are advised to undergo regular follow-up. Therefore, preoperative risk stratification may provide supportive information for treatment planning and perioperative evaluation.

Over the past few decades, risk assessment of gastrointestinal stromal tumors (GIST) has primarily relied on postoperative pathological indicators, including tumor size, mitotic index, and anatomical location. However, these methods are inherently limited by the availability and representativeness of surgical or biopsy specimens. Currently, contrast-enhanced computed tomography (CT) is the standard approach for the preoperative risk stratification of GIST because it enables comprehensive evaluation of tumor size, density, necrosis, and other morphological characteristics. Nevertheless, this technique depends heavily on contrast agent administration, which restricts its applicability in patients with contraindications to contrast media.

In recent years, an increasing number of studies have examined the relationship between visceral fat and tumor development, and a substantial body of evidence indicates that visceral fat is associated with tumor biology, systemic inflammation, and metabolic alterations (1–3). Liu et al. (4) demonstrated that the clinical and pathological characteristics of patients with gastrointestinal stromal tumors (GIST) are significantly associated with lipid levels. To some extent, lipid profile information may serve as a supplementary indicator to differentiate benign from malignant GISTs. The present study was not intended to replace tumor-based imaging analysis, which remains central to GIST risk assessment. Instead, we explored whether visceral adipose tissue may provide additional host-related metabolic and inflammatory information for preoperative evaluation.

Given the inherent density difference between visceral adipose tissue and surrounding structures, visceral fat boundaries can be reliably delineated even on non-contrast CT scans. To enable patients with contraindications to contrast-enhanced imaging to benefit, in the initial phase of our study, we utilized intra-abdominal fat volume to assess gastrointestinal stromal tumors (GIST) and found a positive correlation between intra-abdominal fat volume and GIST risk. However, the sensitivity and accuracy of this approach remain limited and require further improvement (5).

Radiomics, which involves the extraction of quantitative features from medical images, has become a promising tool for characterizing tumors and evaluating their prognosis. Many studies have highlighted the importance of radiomic features in predicting the risk of gastrointestinal stromal tumors (GISTs), assessing treatment responses, and predicting survival outcomes (6, 7). However, traditional radiomics methods face several challenges, including issues with feature reproducibility, subjective feature selection, and limited applicability across different populations. By contrast, recent advancements in deep learning techniques have demonstrated superior performance in analyzing medical images, primarily because these methods can automatically extract high-dimensional features without requiring manual input (8). Despite these benefits, the application of deep learning to the development of clinically relevant prediction models for GISTs has not been thoroughly investigated.

This study aimed to explore whether visceral fat-derived imaging features from non-contrast CT may provide complementary information for preoperative GIST risk assessment, particularly in situations where contrast-enhanced imaging is unavailable or contraindicated. Moreover, we systematically compared this model with traditional radiomic models, deep learning radiomic models, and feature fusion methods to comprehensively evaluate its predictive ability.

## 2 Patients and methods

### 2.1 Study participants

**Figure 1:**
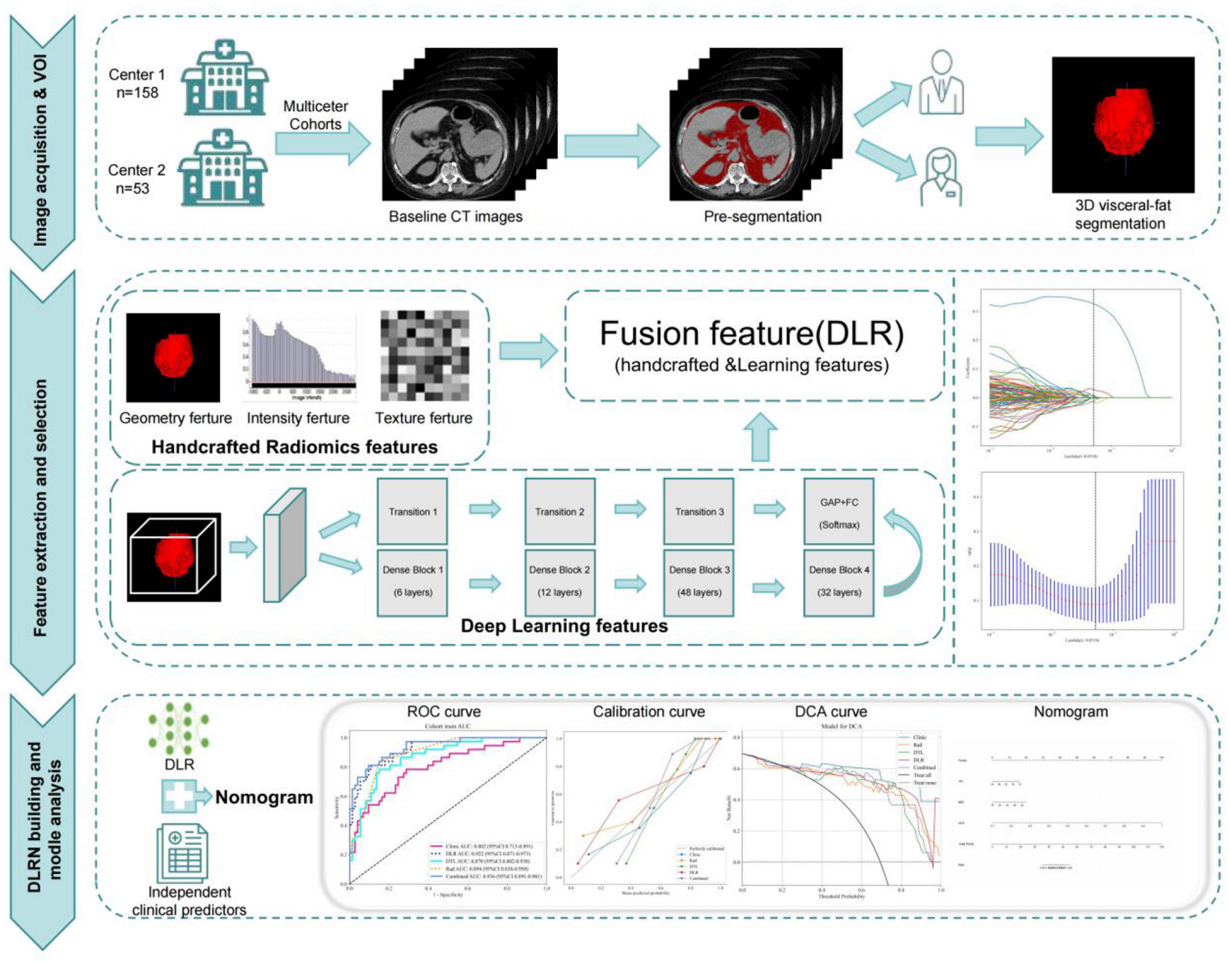
Overall study flowchart. ROC, receiver-operator characteristic; DCA, decision curve analysis.

The clinical and pathological data of patients diagnosed with gastrointestinal stromal tumors (GISTs) who underwent comprehensive abdominal computed tomography (CT) examinations between January 1, 2018, and December 31, 2024, at two medical institutions were retrospectively analyzed. Inclusion criteria were as follows: Cases that met the “China Consensus on the Diagnosis and Treatment of Gastrointestinal Stromal Tumors” diagnostic criteria and were confirmed by pathological examination and had not received any relevant treatment prior to enrollment. Patients who underwent a full abdominal CT scan before surgery, with clear CT images and complete clinical data. The exclusion criteria were as follows: Presence of other metastatic tumors or concurrent primary malignancies. CT images with artifacts or poor gastric cavity filling, resulting in unclear display of the lesion. Subjects who had received any anti-cancer or other treatments that may cause changes in physical composition before surgery. Data from the electronic medical record system were reviewed and collected for this study. The study design and pipeline are illustrated in Figure 1. This retrospective study was approved by the institutional review board of The Third Affiliated Hospital of Chongqing Medical University (IRB: 202301-84). The external validation cohort consisted of retrospectively collected de-identified imaging data obtained through institutional collaboration, and the requirement for informed consent was waived. All methods were performed in accordance with relevant guidelines and regulations.

Finally, 211 patients with GISTs from two independent institutions were included and assigned to two cohorts. The derivation cohort consisted of 158 patients from institution A, who were randomly assigned to a training cohort (TC, *n* = 110) and an internal validation cohort (IVC, *n* = 48) at a ratio of 7:3. The external test cohort comprised 53 patients from institution B and was used to externally validate the model.

### 2.2 Grouping criteria

According to the modified NIH criteria, GISTs were categorized into very low-, low-, intermediate-, and high-risk groups based on tumor size, mitotic activity, tumor location, and tumor rupture (9). Based on the need for adjuvant imatinib therapy, the 211 patients were dichotomized into an intermediate–high-risk group, including intermediate-risk and high-risk GIST cases, and a low-risk group, including very low-risk and low-risk cases.

### 2.3 Image acquisition and delineation of areas of interest

The patients from the two institutions underwent similar CT scan setups using different systems and parameters. The patients were placed in the supine position, and the scan range included the entire abdomen. CT images of the transverse plane were retrieved from a picture archiving and communication system (PACS) and used in this study. The details of CT image acquisition are presented in Supplementary S1. In this study, image annotation and volume of interest (VOI) delineation were performed by two radiologists, each with more than 5 years of experience. The VOIs were manually generated and defined according to the volume of visceral fat. The external test cohort was collected independently at Institution B using a different CT system. The key acquisition parameters for both institutions are summarized in Supplementary S1, including the scanner vendor/model, tube voltage, tube current modulation, slice thickness/increment, reconstruction kernel, matrix size, and field of view. To mitigate potential inter-scanner variability, all VOIs were resampled to an isotropic voxel spacing of 1 × 1 × 1 mm3 and intensity values were normalized to [−1, 1] using min–max normalization before feature extraction. These preprocessing steps were intended to reduce voxel-spacing and intensity-scale variability; however, they were not expected to fully eliminate differences related to anatomical coverage, field of view, or VOI delineation extent.

### 2.4 Image preprocessing

During the training and testing phases, preprocessing was restricted to normalization only, without applying any augmentation. The smallest voxel of VOI was standardized to 1 × 1 × 1 mm^3^. The grayscale values of the image slices were normalized using a min-max transformation to adjust the range to [−1, 1].

### 2.5 Radiomics features extraction

We proposed a DenseNet201-based deep transfer learning (DTL) model to predict the risk grade of gastrointestinal stromal tumors (GISTs) from visceral fat. The architecture of DenseNet201 consists of four densely connected convolutional blocks (Dense Blocks) interleaved with three transition layers. The network begins with a 7 × 7 convolutional layer, followed by max pooling for initial feature extraction. The subsequent Dense Blocks contain 6, 12, 48, and 32 convolutional layers, respectively, each of which establishes dense connections by concatenating feature maps from all preceding layers. This design facilitates efficient feature reuse, alleviates the vanishing gradient problem, and reduces parameter redundancy. Transition layers, consisting of 1 × 1 convolutions and 2 × 2 average pooling, were placed between Dense Blocks to compress feature maps and regulate model complexity. Finally, a global average pooling (GAP) layer followed by a fully connected (FC) softmax classifier produces the prediction output. This densely connected architecture enhances gradient flow and enables the extraction of both low- and high-level features, rendering it particularly suitable for medical image analysis. The network processes visceral fat VOIs as input and outputs probability values for GIST risk stratification. Further details regarding model development and the training process are provided in Supplementary S2.

Initially, 1,834 handcrafted radiomic features and 1,920 DenseNet201-derived deep features were extracted from each visceral-fat VOI. To minimize the risk of information leakage, feature reduction and feature selection were performed exclusively within the training cohort. In the handcrafted radiomics branch, reproducibility filtering, univariable testing, correlation analysis, recursive redundancy removal, and LASSO regression were applied sequentially to obtain the final handcrafted feature subset. In the deep-learning branch, PCA was first fitted in the training cohort to reduce the 1,920-dimensional DenseNet201 feature vector to 512 dimensions, after which the same downstream selection strategy was applied. The resulting PCA loadings, normalization parameters, and selected feature sets were fixed and subsequently applied to the internal validation and external test cohorts without refitting.

The Rad signature was constructed from the selected handcrafted features, whereas the DTL signature was constructed from the selected deep features. For the fused DLR signature, selected handcrafted and deep-learning-derived features were combined and then subjected to additional feature selection. The final DLRN incorporated the fused DLR signature together with selected clinical variables. The final feature composition is summarized in Supplementary S3.

DenseNet201 was initialized with weights pre-trained on ImageNet. Input images were resized to 224 × 224 × 3 to match the network requirements, with single-channel CT images replicated across three channels to form pseudo-RGB inputs. The final classification layer was replaced with a fully connected layer of two nodes for binary risk prediction (low-risk vs. intermediate–high-risk). After replacement of the final classification layer, the model contained approximately 18.1 million parameters. The model was trained using cross-entropy loss and optimized with the Adam optimizer at an initial learning rate of 1 × 10^−4^. A batch size of 16 was used, and training proceeded for up to 100 epochs with early stopping based on validation AUC and a patience of 10 epochs to prevent overfitting. Random seeds were fixed at 42 to ensure reproducibility.

### 2.6 Feature selection and signatures construction

Feature selection was performed in the derivation cohort. For handcrafted features, to ensure the stability of the handcrafted features extracted from the ROI, a random sample of 50 patients was selected, and ROI segmentation was performed twice by two independent radiologists. The inter-class correlation (ICC) coefficient was calculated for each feature. Features with an ICC of 0.9 or higher were considered robust and were retained. To identify the features that were most correlated with risk classification, we performed a *t*-test to select features with significant differences (*p* < 0.05) between the two risk groups. Features with high repeatability were assessed using the Pearson correlation coefficient, which calculates the correlation between features. If any two features had a correlation coefficient greater than 0.9, one feature was retained. To ensure the maximum feature representation, a greedy recursive deletion strategy was employed for feature filtering, wherein the feature with the highest redundancy in the current set was iteratively removed. In addition, the least absolute shrinkage and selection operator (LASSO) logistic regression model was used to further reduce the number of features and select the most relevant features for signature construction. The regularization parameter λ was selected using 5-fold cross-validation within the training cohort, based on the minimum cross-validated deviance.

Because the dimensionality of deep learning features was 1,920, we employed principal component analysis (PCA) to balance the features and reduce their dimensionality. By reducing the number of deep learning features to 512 dimensions, we improved the ability of the model to generalize and mitigated the risk of overfitting. In this study, we also investigated a hybrid approach by combining handcrafted radiomics features and deep learning features to improve the model through feature fusion. An early fusion of the features screened using the aforementioned handcrafted method and the deep learning framework was employed to form a comprehensive feature set. Feature selection was then performed using the same process as for the handcrafted features mentioned above. After feature selection, a conventional radiomics signature (RAD) and a deep learning signature (DTL) were developed using handcrafted features and deep learning features, respectively. In addition, a deep learning-based radiomics signature (DLR) was constructed based on the fusion of the two feature types. In our study, we employed a multilayer perceptron (MLP), a type of feed-forward artificial neural network, to construct the signatures. The MLP is widely used as a supervised learning classifier and is particularly effective for complex and nonlinearly separable data compared with other algorithms (10, 11). The optimal hyperparameters for the model were determined using a grid search method combined with 5-fold cross-validation in the derivation cohort.

### 2.7 Construction of DLRN (nomogram)

A nomogram (DLRN) was constructed using multivariable logistic regression by integrating the fused radiomics signature (DLR) with selected clinical variables (e.g., BMI and VFI) to provide an individualized probability of intermediate–high risk. Potential clinical application may be tailored by selecting threshold probabilities according to local practice and patient preferences, consistent with decision curve analysis.

### 2.8 Performance assessment and model comparison

Risk grade prediction in gastrointestinal stromal tumors (GISTs) was evaluated using receiver operating characteristic (ROC) curve analysis, with scalar metrics including the area under the curve (AUC), sensitivity, specificity, accuracy, and F1 score to determine the optimal ROC point. These performance metrics were compared among the three radiomic signatures (Rad, DTL, and DLR) and the radiomic nomogram (DLRN) across all cohorts. Five-fold cross-validation was used within the training cohort for hyperparameter tuning and model selection. After the optimal settings were determined, the final model was retrained on the training cohort and evaluated on the fixed internal validation cohort and independent external test cohort. Therefore, the performance metrics reported in Table 1 correspond to the fixed cohort evaluations rather than cross-validation averages. The clinical utility of the predictive model was further assessed through decision curve analysis (DCA), and calibration curves were constructed to examine the agreement between predicted probabilities and actual outcomes. The overall study workflow and methodology are illustrated in Figure 1.

**Table 1:** Comparison of various prediction models.

Model name	Acc	AUC	95% CI	Sensitivity	Specificity	PPV	NPV	Cohort
Rad	0.827	0.87	0.8020–0.9381	0.784	0.849	0.725	0.886	Train
DTL	0.845	0.894	0.8384–0.9498	0.838	0.849	0.738	0.912	Train
DLR	0.864	0.922	0.8711–0.9734	0.811	0.89	0.789	0.903	Train
Clinic	0.736	0.802	0.7132–0.8914	0.784	0.712	0.580	0.867	Train
Combined	0.873	0.936	0.8907–0.9812	0.811	0.904	0.811	0.904	Train
Rad	0.75	0.761	0.5356–0.9858	0.778	0.744	0.512	0.935	val
DTL	0.813	0.795	0.6356–0.9541	0.556	0.872	0.6	0.895	val
DLR	0.833	0.86	0.7210–0.9998	0.778	0.846	0.538	0.943	val
Clinic	0.833	0.812	0.6746–0.9494	0.667	0.872	0.545	0.919	val
Combined	0.938	0.892	0.7647–1.0000	0.667	1.000	1.000	0.929	val
Rad	0.811	0.755	0.5174–0.9933	0.667	0.83	0.633	0.951	test
DTL	0.83	0.803	0.5363–1.0000	0.833	0.83	0.585	0.975	test
DLR	0.679	0.858	0.7231–0.9932	1.000	0.638	0.561	1.000	test
Clinic	0.774	0.819	0.6520–0.9863	0.833	0.766	0.612	0.973	test
Combined	0.925	0.862	0.6216–1.0000	0.833	0.936	0.625	0.978	test

The metrics in this table were calculated from the final model evaluated on the predefined training, internal validation, and external test cohorts. Five-fold cross-validation was used for hyperparameter tuning and was not reported as the performance estimate in this table.

AUC, area under the curve; PPV, positive predictive value; NPV, negative predictive value; Val, internal validation test; Test, external validation test.

### 2.9 Statistical analysis

Statistical analyses were performed using SPSS software. Statistical significance was set at *P* < 0.05 (two-sided). Continuous variables were presented as mean ± standard deviation and were compared between the low-risk and intermediate–high-risk groups using an independent-samples *t* test or the Mann–Whitney U test, as appropriate. Categorical variables were presented as counts (percentages) and were compared using the chi-square test or Fisher's exact test. To identify clinical factors associated with intermediate–high risk, univariate logistic regression analyses were first conducted, and variables showing evidence of association were subsequently included in a multivariable logistic regression model to determine independent predictors; odds ratios (ORs) with 95% confidence intervals (CIs) were reported (Table 2). Multicollinearity among candidate clinical variables was assessed using variance inflation factors (VIFs). Variables with a VIF > 5 were considered to show relevant multicollinearity and were not entered simultaneously into the final multivariable logistic regression model. Model discrimination was assessed by ROC analysis, with pairwise AUC comparisons between DLRN and comparator models performed using the DeLong test and Bonferroni correction for multiple comparisons. Calibration curves were used to assess the agreement between predicted probabilities and observed outcomes, and decision curve analysis (DCA) was performed to evaluate potential clinical benefit across a range of threshold probabilities.

**Table 2:** Univariable and multivariable analysis of clinical features.

Parameter	Univariate regression analysis				Multivariable regression analysis			
	Odds ratio	OR lower 95% CI	OR upper 95% CI	*P*	Odds ratio	OR lower 95% CI	OR upper 95% CI	*P*
Clinical characteristics	-	-	-	-	-	-	-	-
Age	0.99	0.985	0.995	< 0.05^*^	0.98	0.945	1.016	0.361
Gender	0.646	0.522	0.801	< 0.05^*^	0.351	0.14	0.926	0.068
location	0.646	0.518	0.805	< 0.05^*^	0.628	0.26	1.516	0.385
Volume (mm^3^)	1.001	1	1.002	0.076				
CEA	0.95	0.883	1.021	0.24				
CA199	0.94	0.907	0.974	< 0.05^*^	1.034	0.959	1.114	0.468
VFI	0.959	0.944	0.975	< 0.05^*^	0.689	0.607	0.782	< 0.05^*^
BMI	0.973	0.96	0.987	< 0.05^*^	1.61	1.305	1.988	< 0.05^*^
SMI	0.988	0.981	0.994	< 0.05^*^	0.98	0.958	1.002	0.132
BMD	0.996	0.994	0.998	< 0.05^*^	1.003	0.995	1.011	0.53
rupture	1.333	0.645	2.754	0.514				
necrosis	1.476	0.928	2.349	0.168				
DM	0.125	0.022	0.715	0.05	0.104	0.008	1.405	0.153
HBP	0.316	0.146	0.682	< 0.05^*^	0.484	0.145	1.616	0.322
CAD	0.667	0.149	2.992	0.657				

OR, odds ratio. ^*^Statistically significant.

## 3 Results

### 3.1 Baseline characteristics

For clinical decision-making regarding adjuvant imatinib, we further dichotomized patients into a low-risk group (very low + low) and an intermediate–high-risk group (intermediate + high). The distribution of these dichotomized risk groups across the training, internal validation, and external test cohorts is provided in Supplementary S4, and pairwise comparisons are also summarized therein.

A total of 211 patients from two institutions were included in this study. The derivation cohort from Institution A comprised 158 patients and was randomly split into a training cohort (*n* = 110) and an internal validation cohort (*n* = 48) in a 7:3 ratio. The external test cohort at Institution B included 53 patients. The distribution of the dichotomized risk groups was as follows: training cohort, low-risk, *n* = 73; intermediate-high-risk, *n* = 37; internal validation cohort, low-risk, *n* = 39; intermediate-high-risk, *n* = 9; and external test cohort, low-risk, *n* = 47; intermediate-high-risk, *n* = 6. The baseline characteristics of the derivation and external cohorts are summarized in Tables 2, 3, respectively. Univariate and multivariate logistic regression analyses were performed to evaluate the associations between clinical variables and intermediate-high risk, and the results are summarized in Table 2. Before multivariable modeling, multicollinearity among candidate variables was assessed using VIFs; variables with VIF > 5 were not simultaneously included in the final model. During VIF-based screening, height and weight were excluded because of collinearity with BMI and other body-composition variables. The final multivariable model retained age, sex, location, CA199, BMI, VFI, SMI, BMD, DM, and HBP. No retained variable showed a VIF > 5.The retained variables showed no severe multicollinearity. Several baseline characteristics differed significantly across cohorts, including fat-volume-related measurements, BMD, tumor location, necrosis, rupture, and CAD (Table 3). These differences indicate the presence of inter-center distributional heterogeneity between the derivation and external test cohorts. Therefore, the external test cohort should be interpreted not only as an independent validation set but also as a cohort with distinct clinical and imaging characteristics.

**Table 3:** Baseline characteristics.

Parameter	Center 1 (*n* = 158)		Center 2 (*n* = 53)	*P*
	Training test (*n* = 110)	Internal test (*n* = 48)	External test (*n* = 53)	-
Clinical characteristics	-	-	-	-
Age	61.71 ± 11.40	61.25 ± 13.98	61.17 ± 11.00	0.954
BMI	23.31 ± 3.11	23.22 ± 3.29	23.13 ± 2.91	0.942
Visceral fat volume (mm3)	193988.16 ± 301587.36	193022.72 ± 391992.786	9405.57 ± 23962.05	< 0.05^*^
BMD	144.91 ± 54.16	150.79 ± 61.89	178.80 ± 52.69	< 0.05^*^
SMI	48.78 ± 26.20	47.06 ± 25.65	48.35 ± 26.74	0.930
VFI	20.80 ± 5.25	20.98 ± 5.86	21.92 ± 5.61	0.470
CA199	8.18 ± 7.15	12.99 ± 23.33	10.85 ± 9.93	0.095
CEA	2.64 ± 4.54	3.16 ± 3.50	2.12 ± 2.00	0.390
Gender				0.240
Female	55 (50.00)	20 (41.67)	31 (58.49)	
Male	55 (50.00)	28 (58.33)	22 (41.51)	
Location				< 0.05^*^
Gastrointestinal tract	60 (54.55)	21 (43.75)	18 (33.96)	
Others	50 (45.45)	27 (56.25)	35 (66.04)	
Necrosis				< 0.05^*^
No	58 (52.73)	26 (54.17)	51 (96.23)	
Yes	52 (47.27)	22 (45.83)	2 (3.77)	
Rupture				< 0.05^*^
No	89 (80.91)	40 (83.33)	52 (98.11)	
Yes	21 (19.09)	8 (16.67)	1 (1.89)	
DM				0.382
No	101 (91.82)	42 (87.50)	45 (84.91)	
Yes	9 (8.18)	6 (12.50)	8 (15.09)	
CAD				< 0.05^*^
No	105 (95.45)	41 (85.42)	44 (83.02)	
Yes	5 (4.55)	7 (14.58)	9 (16.98)	
HBP				0.199
No	85 (77.27)	40 (83.33)	47 (88.68)	
Yes	25 (22.73)	8 (16.67)	6 (11.32)	

^*^Statistically significant. Volume refers to visceral fat volume measured from the segmented abdominal fat VOI and is reported in mm3.

### 3.2 Predictive performance of three radiomics signatures and nomogram

**Figure 2:**
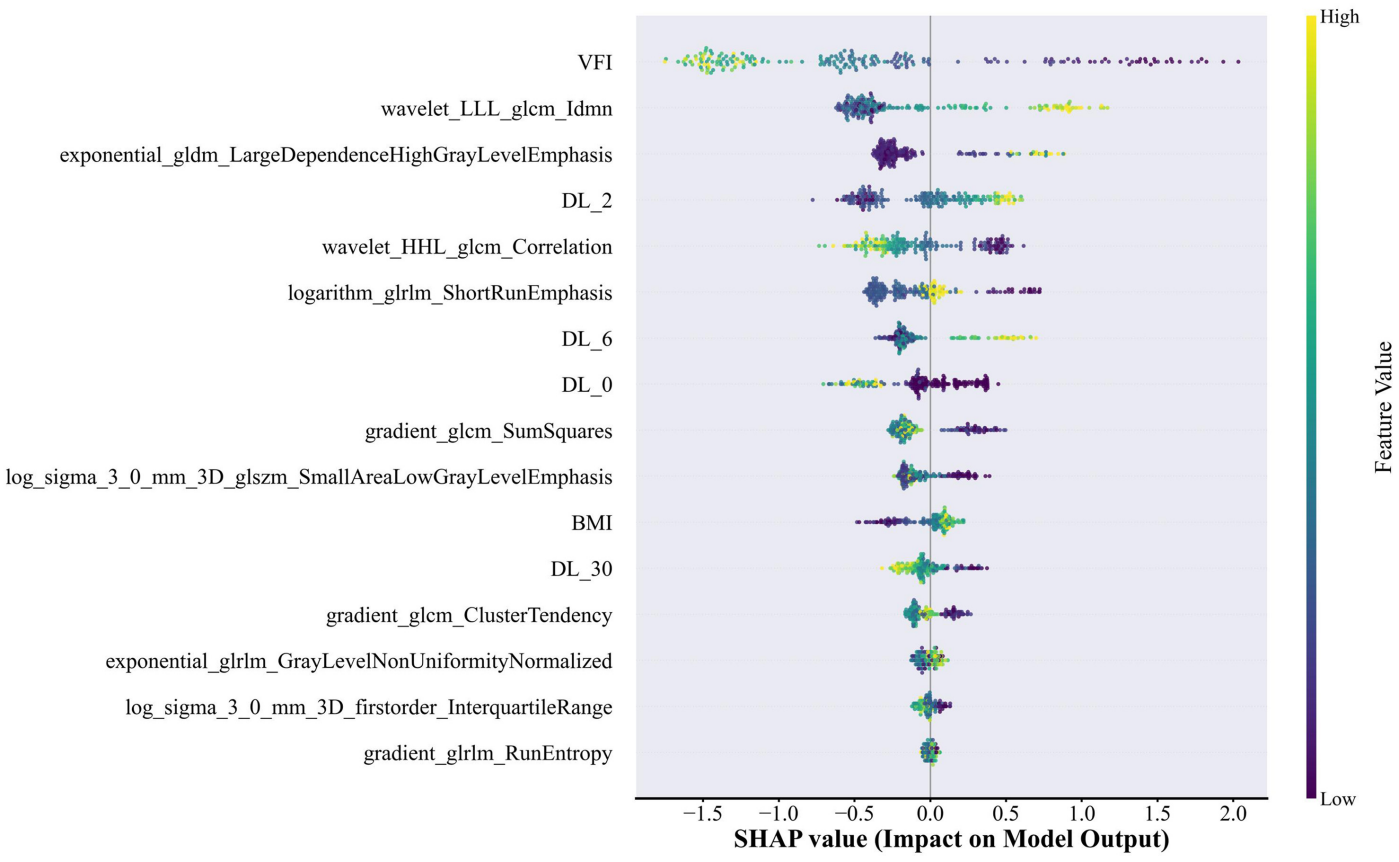
*SHAP summary plot of the selected predictors in the proposed model*. Each point represents an individual patient, with the x-axis indicating the SHAP value and the y-axis showing the selected predictors. The color gradient reflects the feature value, with warmer colors indicating higher feature values. Features are ranked according to their overall contribution to the model output.

**Figure 3:**
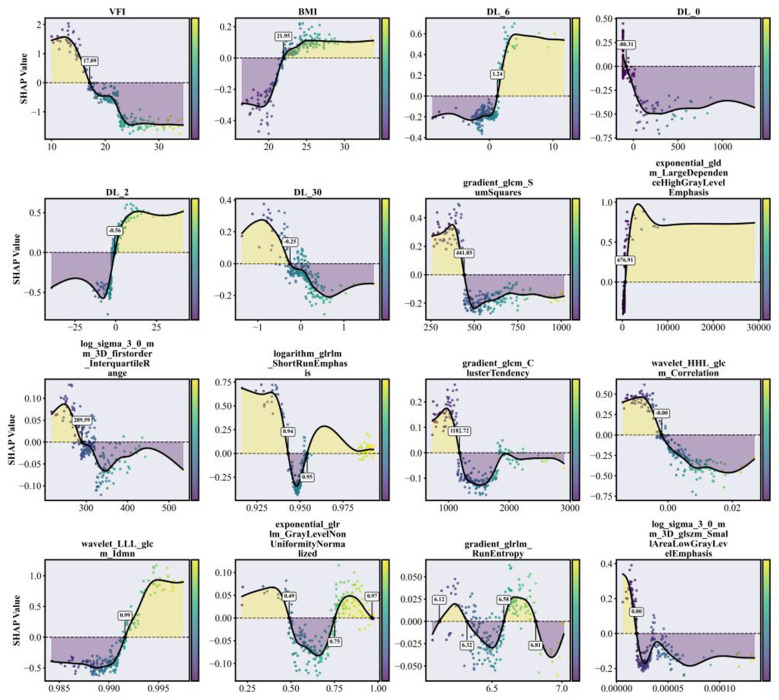
*SHAP dependence plots for the selected predictors in the proposed model*. Each panel shows the relationship between a selected feature and its SHAP value, illustrating the direction and magnitude of its contribution to intermediate–high-risk prediction.

Regarding the fused features, a total of 16 variables were retained for downstream analysis and DLRN development, including 10 handcrafted radiomic features, four deep learning-derived features, and two clinical variables. Figure 2 presents the SHAP summary plot of these selected predictors, showing their overall contribution to the model output. Each point represents an individual patient, with the x-axis indicating the SHAP value and the color gradient reflecting the corresponding feature value. Figure 3 further illustrates the feature-specific SHAP dependence patterns, in which the x-axis represents the feature value and the y-axis represents its SHAP contribution to intermediate–high-risk prediction. The solid black curve shows the smoothed SHAP effect across the feature range, and the horizontal dashed line indicates zero contribution. Positive SHAP values increase, whereas negative SHAP values decrease, the predicted probability of intermediate–high risk. Feature interactions were further explored using an interaction matrix and pairwise SHAP dependence plots, illustrating the joint effects of key predictors on the model output (Supplementary Figure S1).

**Figure 4:**
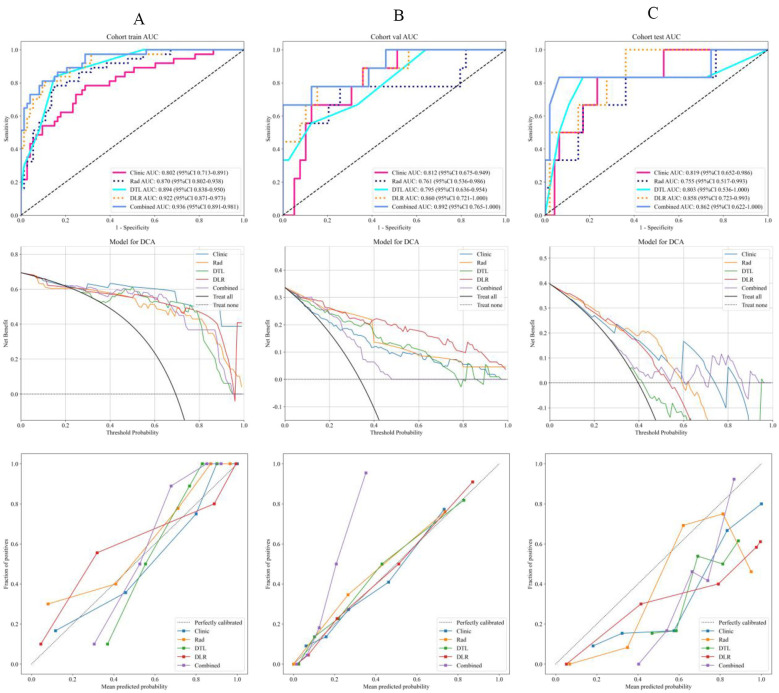
The receiver operating characteristic (ROC) curves, decision curve analysis (DCA), and calibration plots of the clinical, radiomic, deep transfer learning, and combined models in the training cohort **(A)**, internal validation cohort **(B)**, and external test cohort **(C)**, respectively.

**Figure 5:**
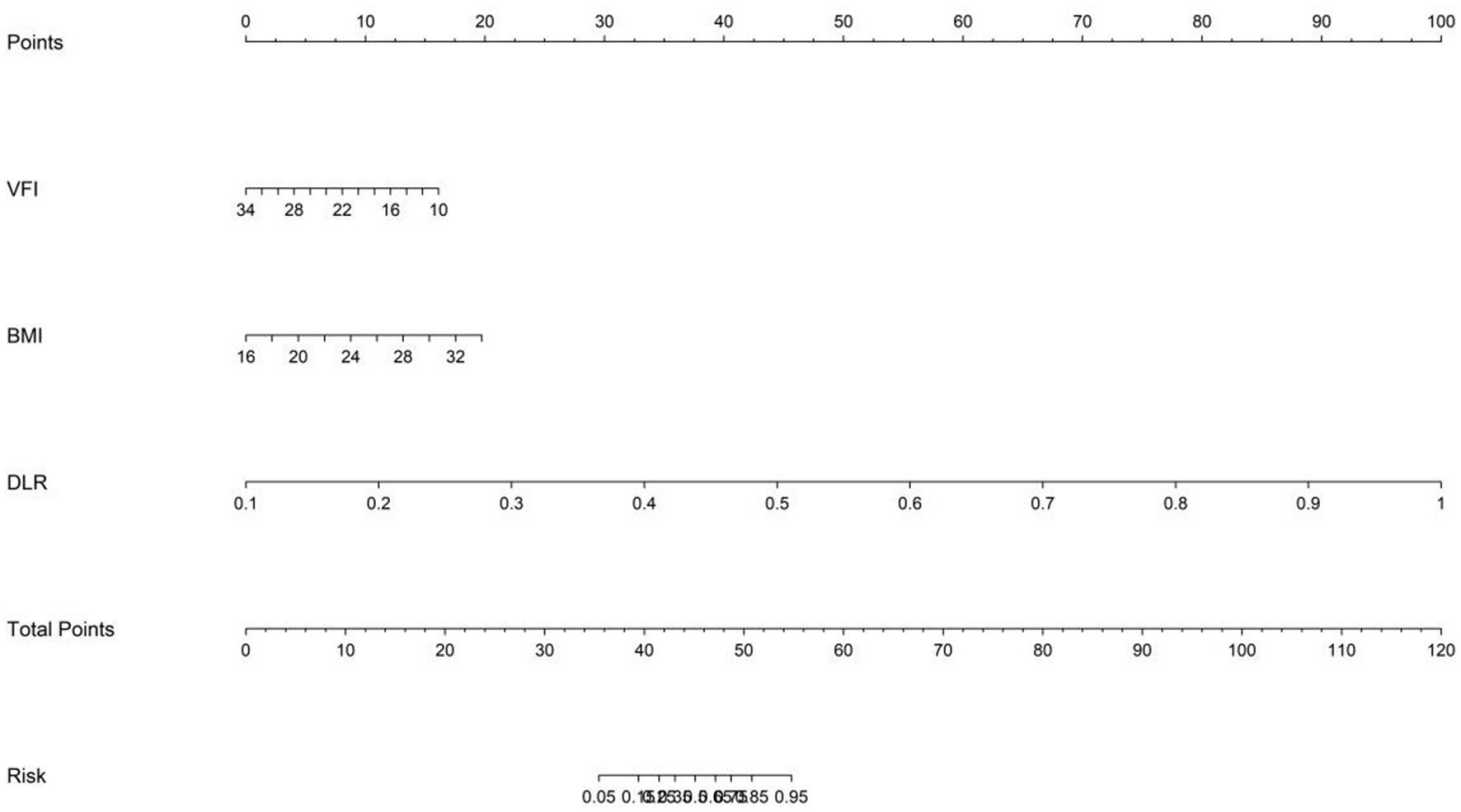
Construction of the deep learning-based radiomic nomogram (DLRN) by integrating the deep learning radiomics signature (DLR) with body mass index (BMI) and visceral fat index (VFI) for risk stratification of gastrointestinal stromal tumors (GISTs).

Table 1 presents a comprehensive overview of the scalar metrics associated with the various predictive signatures and DLR in both the derivation cohort and the external test cohorts. As illustrated in Figure 4, the DTL model achieved an external test AUC of 0.803 (95% CI, 0.5363–1.0000), surpassing the performance of the traditional Rad based on handcrafted features. Following the integration of handcrafted features with deep learning attributes, DLR demonstrated enhanced performance with an AUC of 0.922 (95% CI, 0.8711–0.9734) for the derivation cohort and an AUC of 0.858 (95% CI, 0.7232–0.9932) for the external test cohort. Ultimately, a Deep Learning Radiomics Network (DLRN), which combines radiomic features from DLR with clinical and body-composition variables including BMI and VFI, was developed to visualize individualized risk stratification for GISTs (Figure 5). Among the radiomics-based models, the DLRN achieved the highest performance in the training cohort, with an AUC of 0.936 (95% CI, 0.8907–0.9812), accuracy of 0.873, sensitivity of 0.811, and specificity of 0.904. In the independent external test cohort, the DLRN showed exploratory performance, with an AUC of 0.862 (95% CI, 0.6216–1.0000), accuracy of 0.925, sensitivity of 0.833, and specificity of 0.936. Given that only six intermediate–high-risk cases were included in the external test cohort, these estimates should be interpreted cautiously. To better reflect statistical uncertainty in the imbalanced external cohort, 95% CIs for the scalar performance metrics were additionally calculated and are provided in Supplementary S5.

For both the derivation and test datasets, decision curve analysis indicated that the DLRN provided greater clinical benefit compared with traditional radiomics signatures, deep learning radiomics models, and feature fusion approaches (Figure 4). In addition, calibration curves showed the agreement between predicted probabilities and observed outcomes. Pairwise DeLong tests were performed to compare the AUC of the DLRN with those of the Rad, DTL, DLR, and clinical models, and the results are summarized in Supplementary S6.

## 4 Discussion

Gastrointestinal stromal tumors (GISTs) are the most common mesenchymal tumors of the gastrointestinal tract. These tumors exhibit heterogeneous biological behaviors, with low-risk subtypes typically showing indolent growth, whereas high-risk subtypes are associated with a substantial potential for recurrence and metastasis. Given the distinct therapeutic strategies required for different risk categories (12), accurate preoperative risk stratification is essential to guide clinical decision-making, optimize postoperative surveillance, and improve patient outcomes.

Recent studies have demonstrated that the biological characteristics of visceral fat are closely associated with tumor initiation, progression, and prognosis (13–15). Moreover, owing to the high intrinsic contrast between adipose tissue and surrounding structures, the boundaries of visceral fat can be clearly delineated on computed tomography (CT) images. Therefore, we explored a method based on visceral fat to serve as a new auxiliary approach for preoperative risk stratification of gastrointestinal stromal tumors (GISTs). It should also be emphasized that visceral adipose tissue is not intended to replace tumor-based imaging assessment. Tumor morphology and intratumoural heterogeneity remain the primary determinants of GIST biological behavior. In the present study, visceral fat was evaluated as a complementary host-related imaging biomarker. Direct tumor segmentation on non-contrast CT is feasible and may provide more direct information on tumor size, morphology, necrosis, and intratumoural heterogeneity, which are closely related to established GIST risk criteria. By contrast, visceral fat is a host-level imaging substrate and may reflect systemic metabolic and inflammatory conditions rather than tumor-intrinsic properties. Therefore, the present study does not demonstrate that visceral fat-derived features are superior to direct tumor radiomics. Rather, visceral fat should be regarded as a complementary source of information. Future studies should compare tumor-based, visceral fat-based, and combined ROI strategies on the same non-contrast CT acquisition to determine whether visceral fat provides whether visceral fat provides additional information beyond tumor-centered imaging.

Compared with tumor-centered imaging features, visceral adipose tissue may reflect broader host-related metabolic and inflammatory conditions rather than direct pathological characteristics of the tumor itself. From this perspective, visceral fat-derived imaging information may have potential value in future prognosis-oriented studies involving recurrence or long-term outcomes. However, further validation with standardized follow-up data is still needed to clarify the prognostic significance of visceral fat-derived imaging features in GIST.

In this study, the AUC value of the deep learning-based radiomics model (DLR) in the external validation cohort was found to be significantly higher than that of the traditional handcrafted feature-based radiomics model, suggesting that deep-learning-derived features may capture additional imaging information beyond conventional radiomics descriptors. These methods may capture additional imaging patterns not reflected by conventional handcrafted features that may go unnoticed using conventional techniques. In addition, the deep learning model reduces the need for human intervention and minimizes subjective bias through automated feature extraction, thereby effectively addressing the limitations of manual feature selection in traditional radiomics models (16, 17).

The external test cohort provided an initial assessment of model transportability across institutions; however, the small cohort size and the limited number of intermediate–high-risk cases substantially constrain the precision of the external performance estimates. In particular, the wide 95% CI of the external AUC indicates uncertainty in performance stability. Therefore, our findings should be regarded as preliminary external validation. In future studies, we will expand the sample size, particularly by increasing the number of intermediate–high-risk cases, to further evaluate model robustness, calibration, and clinical utility. From a planning perspective, if the observed external sensitivity of approximately 0.83 is used as a reference, approximately 50–60 intermediate–high-risk cases would be required to estimate sensitivity with an approximate ±10% precision. Therefore, future external validation should aim to include a substantially larger number of positive cases, ideally within a multicentre cohort with a more balanced risk-group composition, to enable more stable assessment of discrimination, calibration, and clinical utility.

We successfully developed a fused model, referred to as the DLRN, by integrating deep learning features with manually extracted radiomic features, resulting in enhanced predictive performance. The DLRN model achieved an area under the curve (AUC) of 0.936 in the derivation cohort and 0.862 in the external validation cohort, suggesting that feature integration may improve model performance. By combining handcrafted features with those derived from deep learning, the model not only improved accuracy but also addressed the limitations inherent in single-feature approaches. Traditional handcrafted features focus mainly on tumor morphology and density, whereas deep learning techniques facilitate the automated extraction of intricate and subtle imaging patterns. This complementary relationship between the two types of features enables the model to provide a more comprehensive representation of the underlying characteristics of gastrointestinal stromal tumors (GISTs), ultimately enhancing predictive accuracy.

This study observed an association between visceral fat characteristics and GIST risk classification, although the underlying biological relationship remains unclear. The direction of the associations between BMI, VFI, and GIST risk should be interpreted cautiously. In the multivariable setting, these findings may partly reflect the complex correlations among body-composition variables rather than simple independent biological effects. Therefore, these results should be considered exploratory and require validation in larger cohorts. This result aligns with our earlier research (5).

The marked difference in visceral fat volume between centers may not be attributable solely to biological differences in patient composition. Technical factors, including scanner type, slice thickness, reconstruction settings, field of view, anatomical coverage, and potential differences in manual VOI delineation, may also have contributed to this discrepancy. Although isotropic resampling and min–max normalization were applied to reduce voxel-spacing and intensity-scale variability, these procedures cannot fully correct differences in anatomical coverage or segmentation extent. Such center-related technical heterogeneity may affect radiomic feature distributions and should be addressed in future studies through harmonized acquisition protocols, standardized VOI delineation procedures, and, where feasible, phantom-based or statistical harmonization.

In clinical practice, the DLRN may be used as an adjunct to routine preoperative assessment based on non-contrast CT, particularly when contrast-enhanced imaging is unavailable or contraindicated. After visceral fat VOI delineation and standardized preprocessing, the model outputs an individualized probability of intermediate–high risk, which can be summarized as a nomogram score and reported alongside conventional CT findings. Such quantitative risk estimates may help prioritize cases for multidisciplinary discussion, support preoperative counseling and planning, and facilitate risk-adapted follow-up strategies. Calibration analysis suggested agreement between predicted and observed risks, whereas DeLong testing was used only for pairwise comparison of discriminatory performance between models. Therefore, model calibration and AUC comparison were interpreted as distinct aspects of performance evaluation. Consistent with the decision curve analysis, these findings suggest potential clinical value across a range of threshold probabilities; however, prospective multicenter validation and workflow impact studies are still needed before broad clinical adoption. These findings suggest that the proposed model may provide complementary information for preoperative GIST risk assessment, although further validation is still required. Although the AUC decreased in the external validation cohort, the model maintained an acceptable balance between sensitivity and specificity. However, given the small and imbalanced external cohort, these findings should be interpreted cautiously and require further validation in larger multicentre cohorts.

Although satisfactory results were obtained in this study, confirming that intra-abdominal fat can predict the risk grade of GIST before surgery, this study does not suggest that visceral fat can completely replace classic pathological indicators (such as size and mitotic figures). Rather, we emphasize that it is a complementary, easily accessible, and preoperatively assessable macroscopic systemic indicator. It may reflect the “fertile soil” (systemic metabolic-inflammatory state) that contributes to tumor progression, combined with the “seeds” (pathological characteristics of the tumor), may provide a more comprehensive risk assessment.

It should also be noted that the present study focused on pathological risk classification rather than direct clinical outcomes such as recurrence-free survival or overall survival. Therefore, the current model should be interpreted as a preoperative risk-assessment approach rather than a direct prognostic model. We agree that prognosis-oriented analysis may be more clinically meaningful, particularly in studies involving host metabolic characteristics such as visceral adipose tissue. However, sufficiently complete and standardized long-term follow-up data were not consistently available in the current retrospective multicentre cohort, which limited our ability to establish a robust prognostic model. Our next step will be to conduct research based on the relationship between intra-abdominal fat and postoperative complications and prognosis of GIST.

The DLRN model demonstrated promising preoperative risk stratification for GISTs, but several limitations should be noted. The external test cohort was small and imbalanced, resulting in a wide AUC confidence interval that reflects uncertainty in performance stability. Baseline differences between institutions, including fat-volume measurements, tumor location, necrosis, rupture, and CAD, suggest a distribution shift that may have influenced external performance estimates. In addition, the current workflow still requires manual delineation of visceral fat VOIs, which may limit immediate clinical scalability. Although feature reproducibility was assessed using ICC, the present study did not formally quantify the time required for VOI delineation or the inter-observer variability of the segmentation masks themselves. Future studies should expand to larger multicentre cohorts with harmonized data collection, balanced risk-group composition, statistical adjustment or stratified evaluation for center-specific heterogeneity, and systematic assessment of segmentation efficiency and mask-level reproducibility. Semi-automatic or fully automatic fat segmentation may further improve workflow efficiency. The inherent “black box” nature of the model also remains a concern; integrating interpretable AI approaches could enhance transparency and facilitate clinician trust and adoption. Overall, these steps are essential to further validate model robustness, calibration, and potential clinical utility.

## Data Availability

The datasets presented in this study can be found in online repositories. The names of the repository/repositories and accession number(s) can be found in the article/supplementary material.
