# Supplementary material for: A deep learning-based radiomic nomogram derived from visceral fat for early prediction of gastrointestinal stromal tumor risk grade
Source: Front Med (Lausanne). 2026 Jun 19;13:1741436. doi: 10.3389/fmed.2026.1741436 (PMC13327938; doi:10.3389/fmed.2026.1741436)
Supplement: Supplementary file 1 [file Table_1.docx]

**The parameters of the CT machine and the scanning parameters.**

| **Parameter** | **Institution A(Center 1)** | **Institution B(Center 2)** |
| --- | --- | --- |
| Scanning device | 256-row MDCT (Revolution CT, GE) | 128-row MDCT (Brilliance iCT, Philips, Netherlands) |
| Scanning range | Upper edge of liver to end of anus | Upper edge of liver to end of anus |
| Scanning mode | Spiral scanning | Spiral scanning |
| Tube voltage (kVp) | 120kVp | 120kVp |
| Tube current (mAs) | 200-600mAs | 200mAs |
| Rotation time (s/rotation) | 0.5s | 0.4s |
| Pitch | 0.992 | 0.925 |
| Matrix | 512×512 | 512×512 |
| Reconstruction thickness (mm) | 2.5 | 3.0 |
| Reconstruction interval (mm) | 0.8 | 0.8 |

To reduce inter-scanner variability, all visceral-fat VOIs were resampled to an isotropic voxel spacing of 1×1×1 mm³, and image intensities were normalized using min–max scaling to [−1, 1] prior to feature extraction.
