# Supplementary material for: A deep learning-based radiomic nomogram derived from visceral fat for early prediction of gastrointestinal stromal tumor risk grade
Source: Front Med (Lausanne). 2026 Jun 19;13:1741436. doi: 10.3389/fmed.2026.1741436 (PMC13327938; doi:10.3389/fmed.2026.1741436)
Supplement: Supplementary file 2 [file Table_2.docx]

### **Deep Learning Procedure**

#### **Data Preparation**

#### **Crop VOI:** To prepare the data, we selected the slice displaying the largest Volume of Interest (VOI) as the representative image for each patient. To streamline the analysis and minimize background noise, we extracted the smallest enclosing cuboid around the VOI.

#### **Data Augmentation:** Data augmentation involved standardizing the intensity distribution across images using Z-score normalization. These normalized images served as inputs for the deep learning model. During training and test images, preprocessing was limited to normalization to ensure consistency in evaluation.

#### **Model Training**

**Transfer Learning:** In this study, we investigated the performance of prominent networks, including ViT, DenseNet201, and CCT, using transfer learning to enhance the transformer framework-based deep learning network (DLN)-based models. We also conducted comparative analyses to determine the most suitable algorithm for our specific research needs.

**Hyper Parameters:** In our study, to ensure the model's effectiveness across various patient populations with notable variability, we implemented transfer learning. This process involved initializing the model with pre-trained weights from the ImageNet database, enhancing its adaptability to diverse datasets. A critical aspect of our approach was the meticulous adjustment of the learning rate to foster better generalization across datasets. For this purpose, we employed the cosine decay learning rate strategy, defined as follows:

$$\eta_{t}=\eta_{min}^{i}+\frac{1}{2}\left( \eta_{max}^{i}-\eta_{min}^{i} \right)\left( 1+cos\left( \frac{T_{cur}}{T_{i}}\pi\right) \right) \text{(1)}$$

The notation $\eta{\text{min}}^{i} = 0$ sets the minimum learning rate, whereas $\eta{\text{max}}^{i} = 0.01$ establishes the maximum learning rate. The term $T_i = 30$ represents the number of epochs in the iterative training process. Additional crucial hyperparameters include the use of Stochastic Gradient Descent (SGD) as the optimizer and the softmax cross-entropy function for the loss.
