# Supplementary material for: A deep learning-based radiomic nomogram derived from visceral fat for early prediction of gastrointestinal stromal tumor risk grade
Source: Front Med (Lausanne). 2026 Jun 19;13:1741436. doi: 10.3389/fmed.2026.1741436 (PMC13327938; doi:10.3389/fmed.2026.1741436)
Supplement: Supplementary file 3 [file Table_3.docx]

**Supplementary Table S3. Feature-selection pipeline and final feature composition**

| **Model** | **Initial feature pool** | **Selection procedure** | **Final input variables / composition** |
| --- | --- | --- | --- |
| Rad | 1,834 handcrafted radiomic features | ICC reproducibility filtering -> univariable testing -> Pearson correlation filtering -> recursive redundancy removal -> LASSO | [n = 518] handcrafted radiomic features |
| DTL | 1,920 DenseNet201-derived deep features | Feature selection performed after PCA using the same downstream selection framework | [n = 512] deep-learning-derived features |
| DLR | Selected handcrafted features + selected deep-learning-derived features | Early feature fusion followed by downstream feature selection | 10 handcrafted radiomic features + 4 deep-learning-derived features |
| DLRN | DLR signature + selected clinical variables | Multivariable logistic regression for nomogram construction | DLR + BMI + VFI / or final verified clinical variables |

**Final feature composition of the fused model and DLRN**

| Feature name | Feature origin | Feature type | Selection stage | Included model | |
| --- | --- | --- | --- | --- | --- |
| VFI | Clinical/body-composition | Clinical variable | Multivariable logistic regression / final DLRN integration | | DLRN |
| wavelet_LLL_glcm_Idmn | Handcrafted radiomics | Texture | Fusion feature selection after handcrafted radiomics filtering and LASSO | | DLR |
| exponential_gldm_LargeDependenceHighGrayLevelEmphasis | Handcrafted radiomics | Texture | Fusion feature selection after handcrafted radiomics filtering and LASSO | | DLR |
| DL_2 | Deep learning-derived | DenseNet201 feature | PCA-reduced deep feature branch followed by downstream feature selection | | DLR |
| wavelet_HHL_glcm_Correlation | Handcrafted radiomics | Texture | Fusion feature selection after handcrafted radiomics filtering and LASSO | | DLR |
| logarithm_glrlm_ShortRunEmphasis | Handcrafted radiomics | Texture | Fusion feature selection after handcrafted radiomics filtering and LASSO | | DLR |
| DL_6 | Deep learning-derived | DenseNet201 feature | PCA-reduced deep feature branch followed by downstream feature selection | | DLR |
| DL_0 | Deep learning-derived | DenseNet201 feature | PCA-reduced deep feature branch followed by downstream feature selection | | DLR |
| gradient_glcm_SumSquares | Handcrafted radiomics | Texture | Fusion feature selection after handcrafted radiomics filtering and LASSO | | DLR |
| log_sigma_3_0_mm_3D_glszm_SmallAreaLowGrayLevelEmphasis | Handcrafted radiomics | Texture | Fusion feature selection after handcrafted radiomics filtering and LASSO | | DLR |
| BMI | Clinical/body-composition | Clinical variable | Multivariable logistic regression / final DLRN integration | | DLRN |
| DL_30 | Deep learning-derived | DenseNet201 feature | PCA-reduced deep feature branch followed by downstream feature selection | | DLR |
| gradient_glcm_ClusterTendency | Handcrafted radiomics | Texture | Fusion feature selection after handcrafted radiomics filtering and LASSO | | DLR |
| exponential_glrlm_GrayLevelNonUniformityNormalized | Handcrafted radiomics | Texture | Fusion feature selection after handcrafted radiomics filtering and LASSO | | DLR |
| log_sigma_3_0_mm_3D_firstorder_InterquartileRange | Handcrafted radiomics | First-order | Fusion feature selection after handcrafted radiomics filtering and LASSO | | DLR |
| gradient_glrlm_RunEntropy | Handcrafted radiomics | Texture | Fusion feature selection after handcrafted radiomics filtering and LASSO | | DLR |
