# Supplementary material for: A deep learning-based radiomic nomogram derived from visceral fat for early prediction of gastrointestinal stromal tumor risk grade
Source: Front Med (Lausanne). 2026 Jun 19;13:1741436. doi: 10.3389/fmed.2026.1741436 (PMC13327938; doi:10.3389/fmed.2026.1741436)
Supplement: Supplementary file 4 [file Table_4.docx]

| **Cohort / Center** | **Total**  **(n)** | **Low-risk**  **(n, %)** | **Intermediate–high risk**  **(n, %)** | **P value*** |
| --- | --- | --- | --- | --- |
| Training cohort (TC) | 110 | 73 (66.4%) | 37 (33.6%) |  |
| Internal validation cohort (IVC) | 48 | 39 (81.3%) | 9 (18.8%) |  |
| External test cohort (ETC) | 53 | 47 (88.7%) | 6 (11.3%) |  |
| **Overall (across cohorts)** | 211 | 159 (75.4%) | 52 (24.6%) | **0.0046** |

| **Comparison** | **2×2 Test** | **P value**  **(two-sided)** | **P value**  **(Bonferroni-adjusted)*** |
| --- | --- | --- | --- |
| TC vs IVC | Fisher’s exact | 0.0857 | 0.2570 |
| TC vs ETC | Fisher’s exact | 0.0023 | 0.0069 |
| IVC vs ETC | Fisher’s exact | 0.4024 | 1.0000 |
