# Supplementary material for: A deep learning-based radiomic nomogram derived from visceral fat for early prediction of gastrointestinal stromal tumor risk grade
Source: Front Med (Lausanne). 2026 Jun 19;13:1741436. doi: 10.3389/fmed.2026.1741436 (PMC13327938; doi:10.3389/fmed.2026.1741436)
Supplement: Supplementary file 5 [file Table_5.docx]

**Supplementary S5. Performance estimates with 95% confidence intervals in the external test cohort**

| **Model** | **Accuracy (95% CI)** | **Sensitivity (95% CI)** | **Specificity (95% CI)** | **PPV (95% CI)** | **NPV (95% CI)** |
| --- | --- | --- | --- | --- | --- |
| DLRN | 0.925 (0.818–0.979) | 0.833 (0.359–0.996) | 0.936 (0.825–0.987) | 0.625 (0.245–0.915) | 0.978 (0.882–0.999) |
