# Supplementary material for: A deep learning-based radiomic nomogram derived from visceral fat for early prediction of gastrointestinal stromal tumor risk grade
Source: Front Med (Lausanne). 2026 Jun 19;13:1741436. doi: 10.3389/fmed.2026.1741436 (PMC13327938; doi:10.3389/fmed.2026.1741436)
Supplement: Supplementary file 6 [file Table_6.docx]

**Supplementary Table S6. Pairwise DeLong tests comparing DLRN with comparator models**

| **Cohort** | **Comparison** | ***p*_value** |
| --- | --- | --- |
| Training | DLRN vs Clinic | 0.008 |
| Training | DLRN vs DLR | 0.628 |
| Training | DLRN vs DTL | 0.002 |
| Training | DLRN vs Rad | 0.012 |
| Internal Validation | DLRN vs Clinic | 0.023 |
| Internal Validation | DLRN vs DLR | 0.029 |
| Internal Validation | DLRN vs DTL | 0.079 |
| Internal Validation | DLRN vs Rad | 0.006 |
| External Test | DLRN vs Clinic | 0.049 |
| External Test | DLRN vs DLR | 0.565 |
| External Test | DLRN vs DTL | 0.8 |
| External Test | DLRN vs Rad | 0.046 |
